# Repeated radiosurgery for recurrent or refractory trigeminal neuralgia: a systematic review and meta-analysis

**DOI:** 10.1007/s10143-025-03853-y

**Published:** 2025-10-18

**Authors:** Federico Valeri, Giuseppe Maria Della Pepa, Fulvio Vincenzo Grilli, Grazia Menna, Nicola Montano, Alessandro Izzo, Pier Paolo Mattogno, Vito Stifano, Silvia Chiesa, Federica Pavoncello, Maria Vittoria Leone, Domenico Lupoi, Federica Murtas, Vincenzo Valentini, Francesco Miccichè, Alessandro Olivi, Francesco Doglietto, Giuseppe Minniti

**Affiliations:** 1 Faculty of Medicine and Surgery, Università Cattolica del Sacro Cuore, Rome, Italy; 2 Department of Neurosurgery, Fondazione Policlinico Universitario Agostino Gemelli, Istituto di Ricovero e Cura a Carattere Scientifico (IRCCS), Rome, Italy; 3 Department of Radiosurgery, Ospedale Isola Tiberina - Gemelli Isola, Rome, Italy; 4 Radiology and Neuroradiology Unit, Department of Imaging, Radiation Therapy and Hematology, Fondazione Policlinico Universitario Agostino Gemelli, Istituto di Ricovero e Cura a Carattere Scientifico (IRCCS), Rome, Italy; 5 Radiation Oncology, Policlinico Umberto I, Department of Radiological, Oncological and Pathological Sciences, Sapienza University of Rome, Rome, Italy

**Keywords:** Radiosurgery, Trigeminal neuralgia, Recurrence, Reirradiation, Stereotactic, Refractory

## Abstract

Stereotactic Radiosurgery (SRS) is a well-established component of the multimodal approach to treating trigeminal neuralgia (TN), a condition marked by frequent and recurrent episodes despite treatments. As a result, an increasing number of centers are offering patients a second or even third round of SRS. Despite this trend, there remains ongoing debate regarding optimal radiation dosing, target planning, overall treatment efficacy, duration of symptom relief, and the safety of repeated procedures. In this study, we present a systematic review and meta-analysis of the existing literature on repeated stereotactic radiosurgery for patients with refractory and recurrent TN. According to PRISMA guidelines, we extracted data regarding general patients features, pre-radiosurgical treatments, irradiation doses of Radiosurgical cycles, target planning, outcome and complications. Out of 461 patients included, 317 (73%) achieved a Barrow Neurological Institute pain scale (BNI) ≤ III after a second SRS procedure. The mean irradiation dose employed in the second treatment was 68.4 Gy with a cumulative dose of 145.9 Gy. Out of the 317 patients with favourable outcome, 101 (31.9%) relapsed after a mean 19.2 months. Patients who responded to a first SRS course were 6 times more likely to achieve pain control after repeateded SRS. Complications were mild and were mainly represented by V cranial nerve dysfunction in 202 patients (43.8%). No cases of radionecrosis, hydrocephalus, or brainstem damage were reported. With a mean follow-up of 19.2 months, repeat SRS was effective in controlling pain in roughly 47% of patients. Dosimetric analysis showed high variability of irradiation doses and target planning. Although data support the safety of repeated SRS in patients with refractory or recurrent TN, homogeneous treatment protocols and uniformity in data reporting is needed to optimize the potential of repeated SRS.

## Introduction

Trigeminal Neuralgia (“TN”) is a condition characterized by paroxysmal episode of unilateral facial pain, often triggered by stimuli or environmental factors, along one or more branches of the V cranial nerve [1].

Patients afflicted by it experience frequent treatment failures and multiple short-term recurrences, and often undergo multiple procedures [2, 3]. TN management poses a significant challenge for the clinicians involved as both surgical and medical treatment fall short in long term control of the condition [4].

The role of Stereotactic Radiosurgery (SRS) is integrated within a multimodal treatment strategy and is often considered a therapeutic option following the failure or intolerance of pharmacological therapy, or after the unsuccessful outcome of etiological surgical interventions such as microvascular decompression (MVD) of the Trigeminal nerve.

Given that the condition is marked by multiple and frequent recurrences, and considering that the therapeutic effect of SRS is often temporary, an increasing number of centers are now referring patients for a second or even third round of radiosurgical treatment. However, ongoing debate remains regarding optimal radiation dosing, target definition, overall efficacy, the expected duration of symptom relief, and the safety of repeated SRS procedures.

A standardized approach is of utmost importance in TN reirradiation as: (1) TN may recur earlier than other pathologies commonly treated with SRS (e.g.: meningiomas, schwannomas); (2) there is little evidence about the effect of repeated SRS cycles on outcome and complications; (3) dose is typically administered the target is small and localized in a space packed with delicate brain and nervous structures which has already received high radiation dose.

The aim of this study was to systematically review and analyze available data on repeated SRS for recurrent or refractory TN to properly individuate its place in the management algorithm of such a complex disorder.

## Methods

**Fig. 1:**
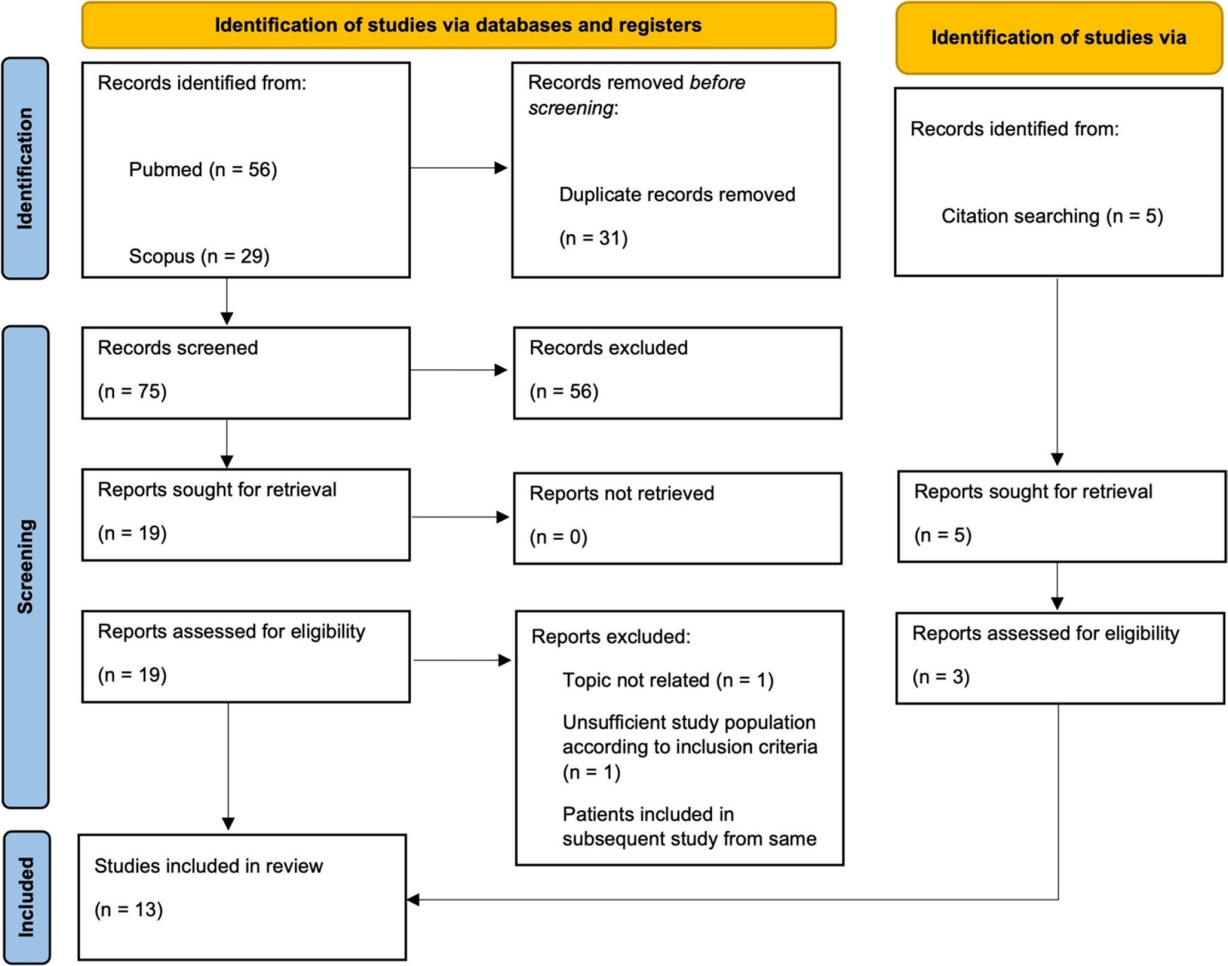
PRISMA 2020 diagram of the systematic review

This review was carried out accordingly with the updated Preferred Reporting Items for Systematic Reviews and Meta-Analysis (PRISMA 2020) guidelines [5] [Fig. 1].

### Research strategy and study selection

A computer aided search of PubMed, Scopus, and Ovid databases was performed in order to identify relevant studies. A search string was developed for each database to extract papers on the use of repeat Radiosurgery in recurrent or refractory TN. A combination of the following search terms was used: “radiosurgery”, “gamma knife”, “cyber knife”, “gammaknife”, “cyberknife”, “stereotactic radiosurgery”, “trigeminal neuralgia”, “recurrence”, “retreatment”, “repeated”, “salvage”, “reirradiation”, “re-irradiation”, “re-treatment”.

All papers published until February 5th 2025 were considered; titles and abstracts were screened independently by two reviewers (FV and FVG) against a set of pre-defined eligibility criteria. We included original studies in English reporting at least 10 patients treated with at least two cycles of SRS for recurrent or refractory TN. To be included, each study had to report: (1) a minimum follow-up of 6 months from the latest treatment; (2) pain outcome after the second SRS; (3) irradiation dosages; (4) complications after SRS.

Eligible studies were screened for full-text analysis, and disagreement were resolved by consensus or appeal to a third senior reviewer (GMDP).

### Data extraction and outcome measures

An Excel sheet was developed to extract data on the following variables: study details, sample size, demographics, TN specifics (typical/atypical, length of symptoms pre-SRS), multiple sclerosis (“MS”), prior treatment, radiosurgery characteristics (technology, dosage, target), pain outcome, recurrence after treatment, time to reirradiation, intervention in between SRS cycles, complications after SRS treatment, and follow-up time.

Primary outcomes measures considered were:

1. Rate of pain control after SRS;
2. Irradiation doses;
3. Assessment of complications after retreatment.

Secondary variables studied were time in-between SRS cycles, and target planning strategies.

Pain was assessed according the Barrow Neurological Institute (“BNI”) pain intensity scale [6] [Table 1]. We defined refractory TN as a persistent BNI IV or V after the first SRS treatment, while recurrent TN was used in cases of relapse of facial pain after an initial BNI ≤ III after the first SRS treatment.

**Table 1:** Barrow neurological Institute (“BNI”) facial pain scale

BNI pain intensity score	Definition
I	No trigeminal pain, no medication
II	Occasional pain, not requiring medication
III	Some pain, adequately controlled with medication
IV	Some pain, not adequately controlled with medication
V	Severe pain/no pain relief

### Statystical analysis

We conducted a meta-analysis based on 13 published studies, encompassing a total of 461 patients who underwent SRS for trigeminal neuralgia. Demographic and clinical characteristics—including sex, age, TN subtype, MS status, prior treatments, and symptom duration—were extracted when available. Treatment parameters such as target-to-brainstem distance, radiation doses for the first, second, and third SRS sessions, and recurrence rates were recorded. Descriptive statistics (mean, standard deviation, range, and proportions) were calculated for continuous and categorical variables. Clinical outcomes were assessed using the Barrow Neurological Institute (BNI) pain scale, categorizing patients into favorable (BNI ≤ III) and unfavorable (BNI > III) response groups. A random-effects meta-analysis was performed to estimate pooled odds ratios (ORs) with 95% confidence intervals (CIs), evaluating the likelihood of achieving a favorable outcome. Between-study heterogeneity was assessed using the I² statistic and chi-square test. A forest plot was created to illustrate individual study estimates, confidence intervals, study weights, and the pooled effect size.

Data were compiled and analyzed using Microsoft Excel (Version 16.78, Microsoft Corporation, Redmond, WA, USA), which was also used to generate summary statistics and prepare data for graphical visualization.

## Results

### Demographics

**Fig. 2:**
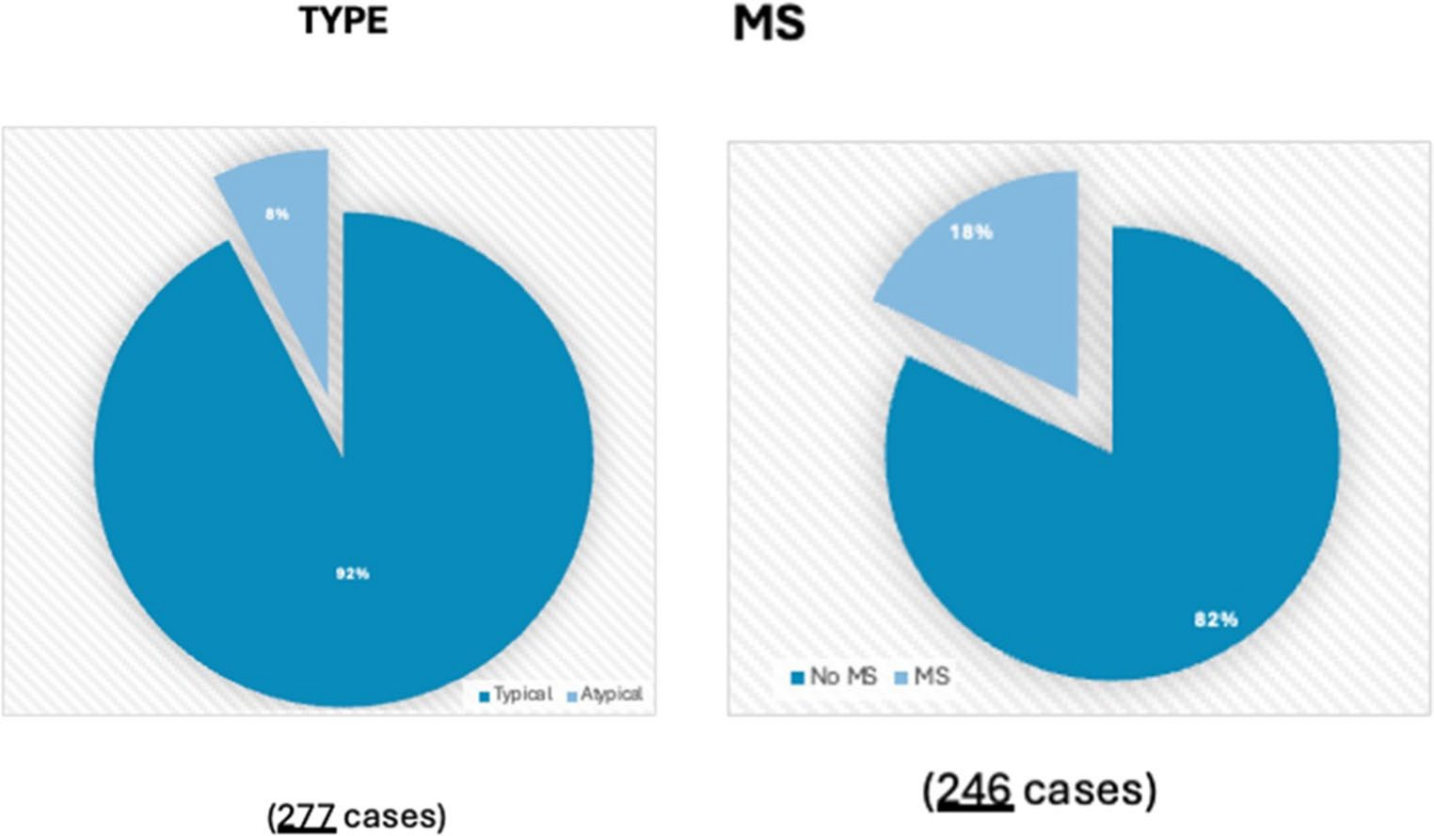
Graphical depiction of TN type and MS diagnosis

Based on the data from 13 studies [7–19], a total of 461 patients who underwent SRS were analyzed. All patients were treated with Gamma Knife SRS (GKSRS). Demographic information was available for most patients. Regarding sex distribution, 59% were female and 41% male (407 cases). Age data, reported in 426 cases, showed a mean age of 67.31 ± 5.9 years. TN type was specified in 277 cases, with 92% of patients classified as typical and 8% as atypical. Multiple sclerosis (MS) status was reported in 246 cases, with 82% of patients not having MS and 18% diagnosed with MS [Fig. 2]. Data on prior treatments was available for 463 patients: 264 (57%) reported no treatment before the first SRS, while 159 (34%) had undergone prior procedures, including rhizotomies (56 patients), MVD (36 patients), radiofrequency (20 patients), GKS (16 patients), and others. The average symptom duration before SRS was 53.4 ± 47 months, based on 224 cases.

The median follow-up after the final SRS session was 24 ± 14.7 months.

Helis series [14] lost 25 patients to follow-up, whose demographical data could not be extracted to exclude it from analysis.

General data is summarized in Table 2.

**Table 2:** Summary of general patients’ data and radiosurgical parameters extracted from studies

General data	
Patients (N)	461
Sex	167/407 Males (41%)
	240/407 Females (59%)
Mean age at last SRS	67.3 (± 5.9) years
Mean pre-SRS Symptoms duration	53.4 (± 47) months
Follow-up after last SRS treatment	24 (± 14.7) months
Radiosurgery parameters	
Mean dose at first SRS	77.5 ± 9.5 Gy (range: 35–90 Gy)
Mean dose at second SRS	67.6 ± 15.1 Gy (range: 35–102 Gy)
Mean cumulative dose	145.9 ± 17.8 Gy (range: 105–180 Gy)
Mean time in-between SRS Cycles	19.6 months (range: 8–45 months)

### Dose

All reported radiation doses refer to the maximum dose at the isocenter. All studies delivered radiation with a Gamma Knife 4 mm cone beam.

The average target-to-brainstem distance was reported in eight studies, encompassing 295 patients, with a mean of 4.3 ± 1.8 mm. The average dose administered during the first SRS was 77.5 ± 9.5 Gy (range: 35–90 Gy), while the second SRS had an average dose of 67.6 ± 15.1 Gy (range: 35–102 Gy). The median cumulative dose from the first and second treatments was 145.9 ± 17.8 Gy. Recurrence following the second SRS occurred in 101 out of 428 patients (23.5%). Among these, 26 patients (9 from Huang [13] and 17 from Tempel [16]) underwent a third SRS, accounting for 25.7% of those with recurrence. The third SRS dose, reported in a single study, averaged 70 Gy (range: 40–80 Gy), with a median cumulative dose of 210 Gy (range: 150–240 Gy). Recurrence after the third SRS was only reported in the same single study (in 4/17 patients).

### Outcome

Clinical outcomes after the first SRS were overall favorable (out of 333 cases with reported outcome, 77% had a BN ≤ III). Among 111 patients for which this variable was reported, the average time to recurrence following the first SRS was 25.9 ± 13.5 months. The time to re-irradiation was reported in 426 patients with a mean interval of 20.9 ± 10.6 months. Prior to undergoing a second SRS, 20 patients (out of 244 for which this variable was reported) had received additional interventions: 12 underwent microvascular decompression (MVD), 7 had rhizotomies, and 1 received radiofrequency treatment.

Regarding the targeting strategy for the second SRS, six studies (181 patients) reported no variations compared to the first treatment, while another study (16 patients) did not discuss the approach used at all. One study (18 patients) applied minimal modifications to minimize overlap. Two studies (44 patients) targeted areas anterior to the initial site with approximately 50% overlap. In 19 patients, the second SRS target was positioned 2–4 mm distally. One study involving 152 patients employed a distal-proximal inversion technique, and in 31 patients, minimal changes were made to ensure the brainstem received a maximum point dose of less than 20 Gy.

**Fig. 3:**
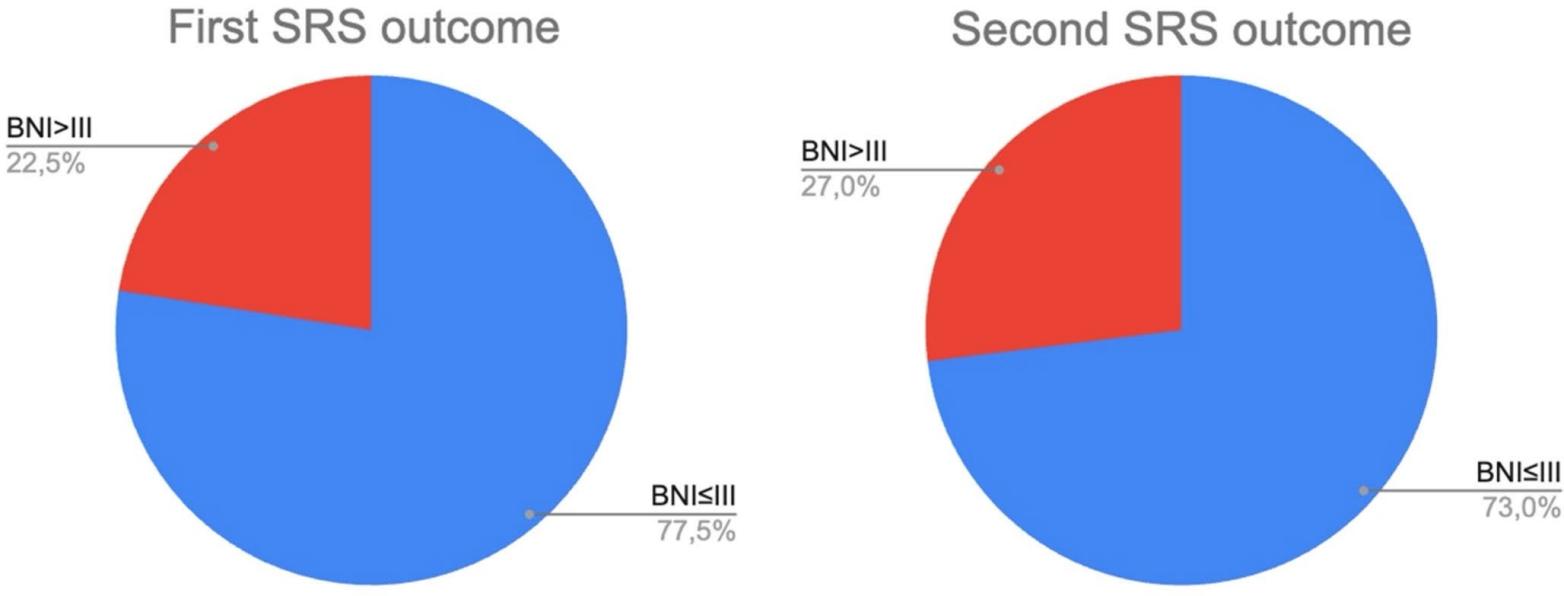
Graphical depiction of outcomes after first SRS (left, 333 cases) and second SRS (right, 434 cases)

Clinical outcomes after the second SRS were reported for 434 patients, comparable to the first treatment (73% BNI ≤ III) [Fig. 3].

Two authors did not report whether their patients underwent some other procedure before SRS [7, 11]. Only 3 reported SRS as their first non-medical treatment [13, 18, 19] for a total of 64 patients. They achieved pain control in 48 patients (75%) after the first SRS cycle, and in 44 patients (64.7%) after a repeated SRS. In the other 8 cases [8–10, 12, 14–17] where other treatment were explored before the first SRS, out of 299 patients where BNI was reported after the first SRS, 227 (75.9%) achieved pain control, and 239 out of 359 (66.6%) after the second SRS.

**Fig. 4:**
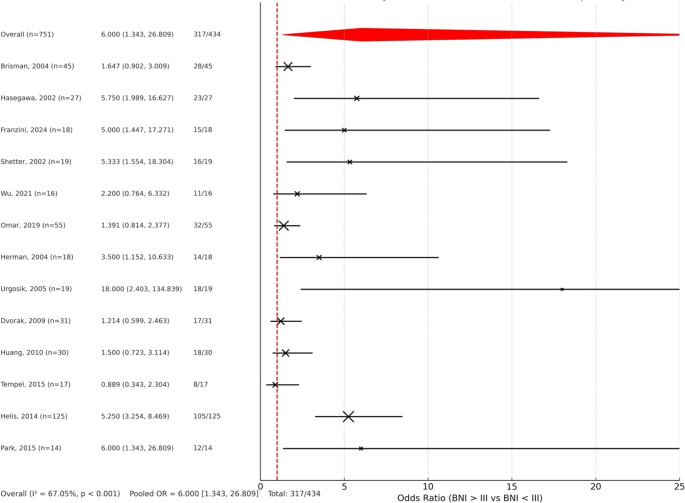
Forrest-plot showing study estimates and pool effect analysis

The meta-analysis included 434 patients from the 13 studied analyzed, evaluating the odds of achieving a favorable outcome (BNI ≤ III) versus an unfavorable one (BNI > III) following treatment. The pooled odds ratio (OR) was 6.00 (CI 95%: 1.3–26.8), indicating that patients were six times more likely to achieve favorable outcomes. The result was statistically significant (*p* < 0.05) and suggests a robust treatment effect. However, there was moderate to substantial heterogeneity among studies (I² = 67.1%, *p* < 0.001), indicating variability across study results. A forest plot was generated [Fig. 4] showing individual study estimates, confidence intervals, and the weighted pooled effect, visually reinforcing the direction and strength of the findings.

Among the 317 patients who responded to the second course of SRS, 101 experienced another relapse (31.9%) after a mean time of 19.2 months (range 2-123 months).

### Complications

**Fig. 5:**
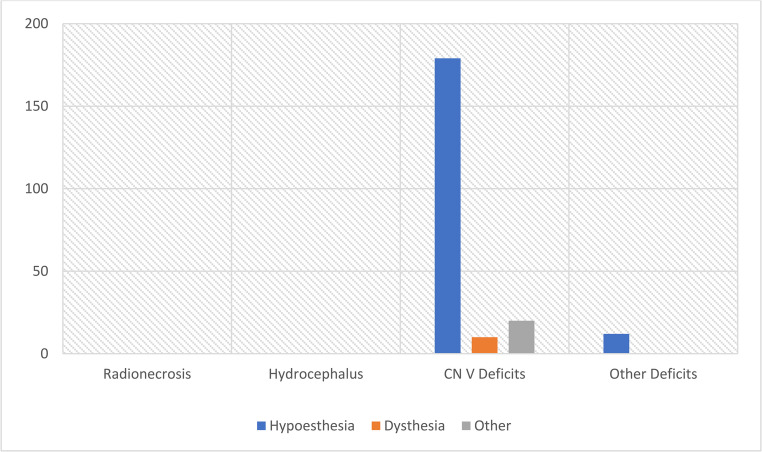
Bar graph of complications after SRS treatment for TNBar graph of complications after SRS treatment for TN

Complications occurred in 221 out of 461 patients (47.9%). The majority of these involved V cranial nerve, affecting 209 patients (45.3%). Among these, hypoesthesia was the most frequently reported symptom (179 cases), followed by dysesthesia (10 cases), while 20 patients experienced other types of trigeminal dysfunction not further specified. An additional 12 patients (2.6%) developed other neurological deficits such as corneal dryness (10 cases), dysgeusia and hypogeusia (one case respectively) [Fig. 5]. Studies did not provide a grading of severity of such complications to properly compare them.

See Table 3 for an extended description of data extracted from each study.

**Table 3:** Extended description of data extracted from studies

Author, year	First irradiation dose (mean, range)	BNI ≤ III after first SRS (*n*, %)	Recurrence time (months)	Reirradiation time (mean)	Second irradiation dose (mean, range)	BNI ≤ III after second SRS (*n*, %)	Target variation between SRS cycles	Recurrence after second SRS (*n*, %)	V cranial nerve deficit (*n*, %)
Shetter, 2002 [7]	78.2 (70–90)	NA	NA	NA	46.6 (35–80)	16 (84%)	No	NA	8 (42%)
Hasegawa, 2002 [8]	75.6 (60–80)	23 (85%)	18.2	22.3	64.4 (50–80)	23 (85%)	Target anterior to first treatment with 50% overlap	4 (14%)	5 (18.5%)
Herman, 2004 [9]	75 (70–80)	14 (78%)	NA	8	70 (65–75)	14 (78%)	No	3 (17%)	2 (11%)
Brisman, 2004 [10]	75 (70–75)	29 (64%)	NA	18	40	28 (80%)	No	11 (24%)	6 (13%)
Urgosik, 2005 [11]	75 (70–80)	NA	21	12.5	75 (70–80)	18 (95%)	2–4 mm distal to first treatment	1 (5%)	6 (31.6%)
Dvorak, 2009 [12]	80 (80–85)	NA	NA	18.1	45 (40–50)	17 (55%)	Minimal changes to ensure brainstem < 20 Gy maximum point dose	7 (23%)	8 (26%)
Huang, 2010 [13]	49 (35–50)	17 (47%)	NA	8	80 (75–102)	18 (60%)	No	9 (30%)	11 (36.6%)
Helis, 2014 [14]	90 (85–90)	127 (84%)	NA	16	80 (80–85)	105 (84%)	Distal/proximal inversion to minimize overlapping between procedures	30 (20%)	101 (80%)
Park, 2015 [15]	85 (70–90)	11 (79%)	54	45	85 (50–90)	12 (86%)	No	NA	5 (36%)
Tempel, 2015 [16]	80 (70–85)	6 (35%)	22.1	34.3	70 (40–80)	8 (47%)	Target anterior to first treatment with 50% overlap	17 (100%)	6 (35%)
Omar, 2019 [17]	75 (70–80)	NA	NA	25.7	75 (70–80)	32 (58%)	No	9 (16%)	25 (45.4%)
Wu, 2021 [18]	85	13 (81%)	28.4	NA	73	11 (69%)	NA	5 (31%)	12 (75%)
Franzini, 2024 [19]	85 (80–85)	18 (100%)	11.5	22.5	85 (75–80)	15 (83%)	Modified to minimize overlap	5 (28%)	7 (39%)

### Risk of bias

**Fig. 6:**
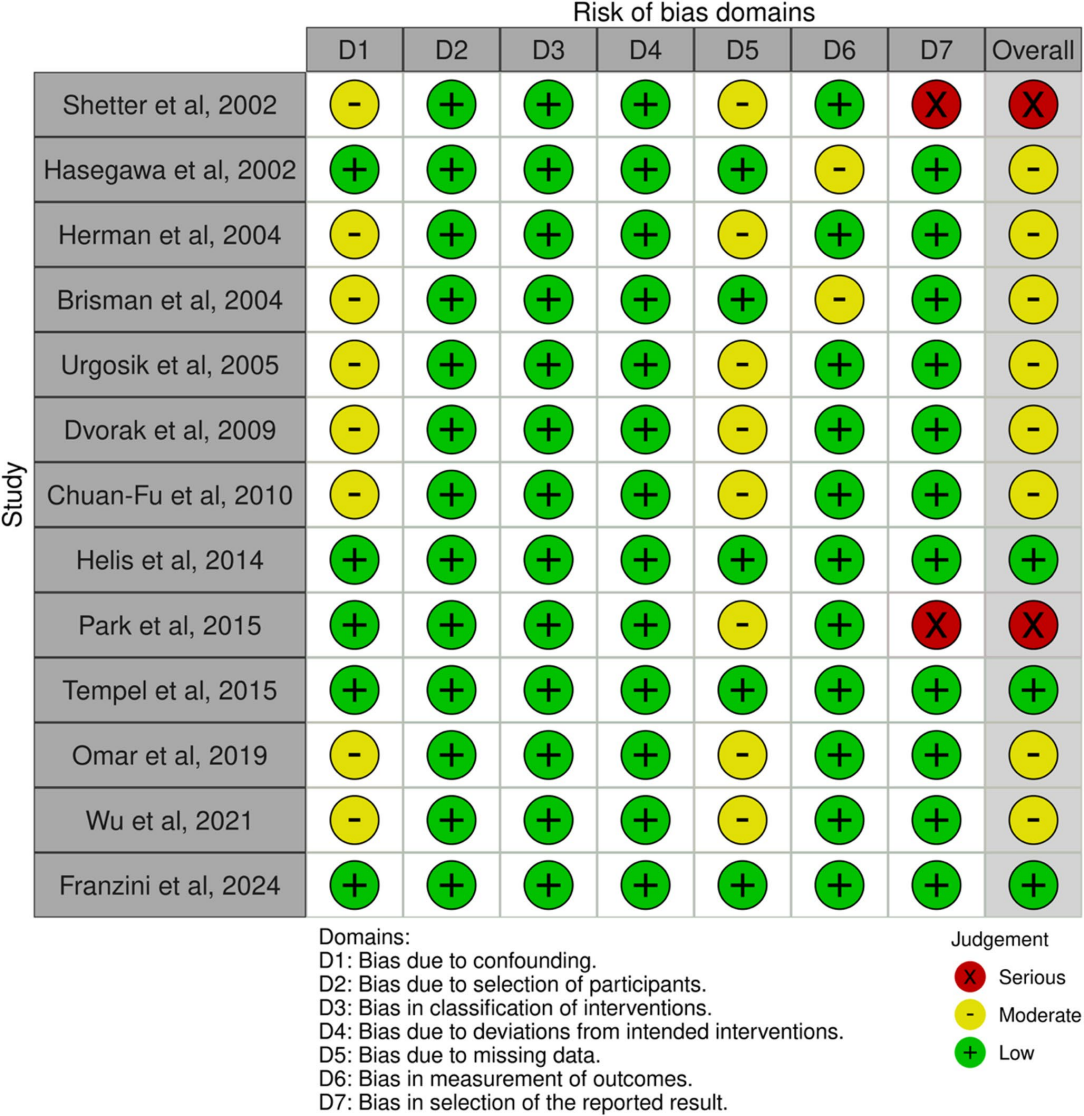
ROBINS-I tool graph for risk of bias assessment

Risk of bias was assessed across all included studies using the ROBINS-I tool [Fig. 6]. Most studies demonstrated a low risk of bias in key domains such as classification of interventions (D3), deviations from intended interventions (D4), and handling of missing data (D5), suggesting consistent methodology and reporting. However, several studies showed moderate risk of bias, particularly in confounding (D1) and selection of participants (D2). Serious risk of bias was identified in the domain of selective reporting (D7) in three studies (Shetter et al. [7], Helis et al. [14], and Tempel et al. [16]), leading to an overall serious risk of bias classification for these studies. Only one study (Franzini et al. 2024 [19]) was rated as having low risk of bias across all domains, indicating the highest methodological rigor among the included papers.

In this meta-analysis, a pooled odds ratio of 6.00 (95% CI: 1.34–26.81) indicates a strong and statistically significant effect, suggesting that the studied exposure or intervention markedly increases the likelihood of the outcome. However, the observed heterogeneity (I² = 67.05%, *p* < 0.001) highlights considerable variability among studies. Complementing this, the funnel plot reveals a notable asymmetry, with a scarcity of studies on the left side of the pooled estimate, raising concerns about potential publication bias. This suggests that smaller studies with non-significant findings may be underrepresented, potentially inflating the overall effect size. Together, these findings underscore the need for cautious interpretation of the pooled results and advocate for broader data inclusion to enhance robustness.

**Fig. 7:**
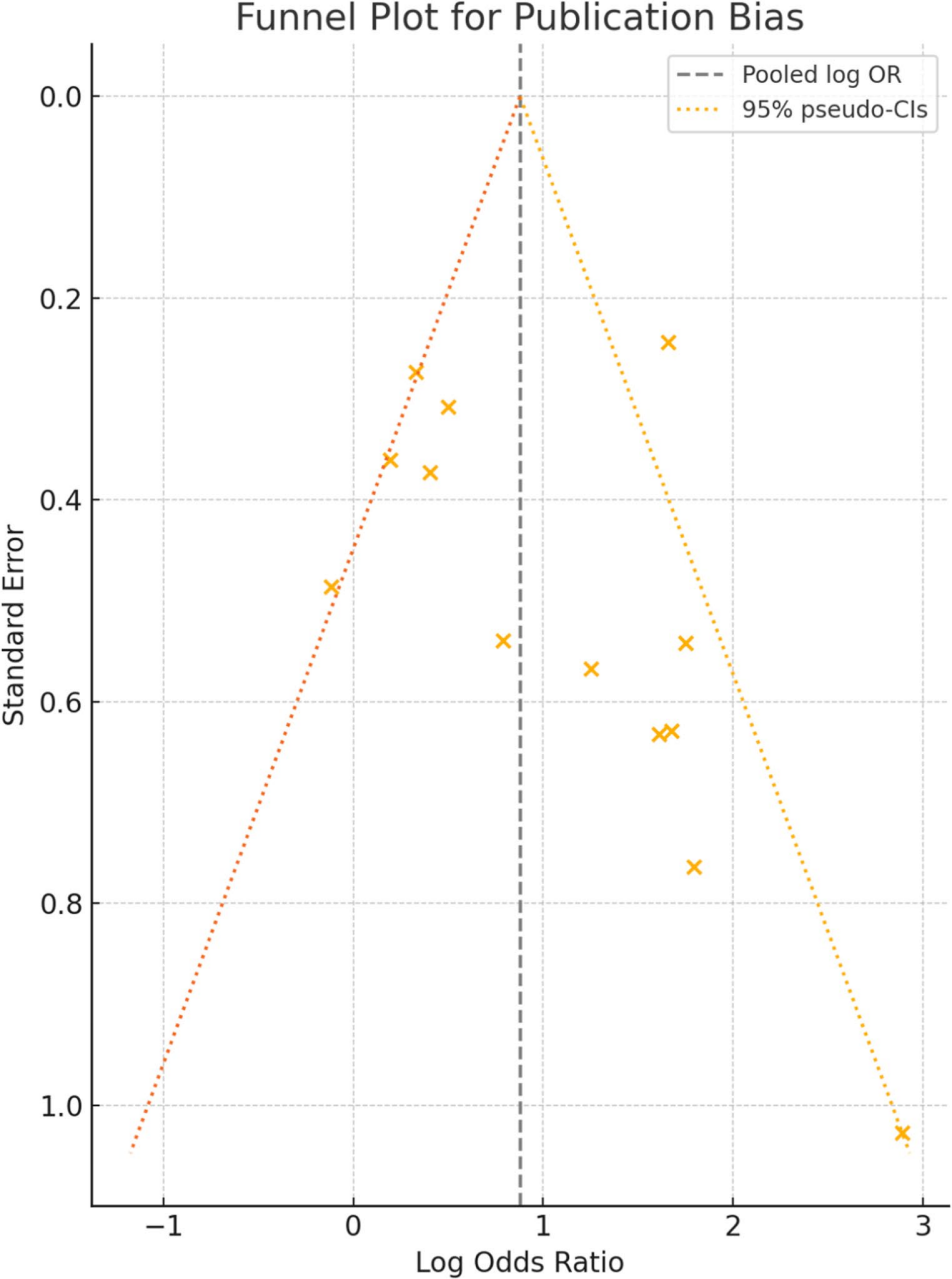
Funnel-plot for Egger’s regression test

We evaluated funnel-plot asymmetry using Egger’s regression test (*p* = 0.639). While visually asymmetric, it did not detect statistically significant publication bias. These findings imply that while the pooled estimate is robust, caution is still warranted due to heterogeneity, though the risk of systematic publication bias appears minimal [Fig. 7].

## Discussion

SRS is known to be effective in achieving pain control in patient with TN [20–22], yet, even when initially successful, up to 52% of patients experience pain relapse post-SRS [23]. Many patients have been referred for a second course of SRS after failure of a first course since the 2000 s; however, there is no general consensus on SRS doses, treatment planning details - such as dose constraint on adjacent organs at risk (OAR) - and overall efficacy and safety of repeat radiosurgery in recurrent or refractory TN.

### Irradiation dosage and target planning

Mean irradiation dosage across studies included was 77.5 ± 9.5 Gy (range: 35–90 Gy) during the first SRS, and 67.6 ± 15.1 Gy (range: 35–102 Gy) for the second SRS, with a median cumulative dose of 145.9 ± 17.8 Gy, and every patient underwent one SRS session. Radiation dosage evidently out-scales that of other benign pathologies of the Central Nervous System. Both radiation dose and target placement are tuned accordingly both to maximize V cn irradiation and to minimize brainstem exposure.

While most authors before 2010 significantly lowered the dose after the first treatment [7, 8, 10, 12] to protect the brainstem – with cumulative doses usually under 140 Gy - this trend hasn’t been followed in the later years [13–19] when authors did not report significant lower dose compared to the first treatment (cumulative doses over 150–170 Gy). This shift in paradigm might be explained by two factors: (1) the increasing confidence in SRS safety through the years due to the absence of radionecrosis reported both before and after 2010; (2) the technological advancement through the years. In particular, all studies included in this review administer SRS via the GammaKnife (“GK”) system, but while first case series of repeat SRS for TN reported the implementation of the GK Model B or U [7, 8], latest research implemented the C, Perfexion, or Icon Model [17, 19].

Regarding target delineation, Wu et al. [18] did not specify if their target varied between first and second cycle of SRS. The remaining authors reported changing their target in 6 cases [8, 11, 12, 14, 16, 19]: in 4 cases [8, 11, 12, 16], the authors placed the target of the second SRS anteriorly to the first one to protect the brainstem, while Franzini et al. [19] and Helis et al. [14] only modified their treatment to minimize overlap between procedures and maximize V cn irradiation. The remaining 6 authors did not change their target [7, 9–17]. While all authors paid attention not to overexpose the brainstem to radiation, only Park et al. [15] reported the cumulative volume of the brainstem that received more than 12 Gy (VB12 = 10.9 ± 3.3 mm^3^). A biological parameter is fundamental to properly assess a standardized planning since there is no consensus on the best target strategy: some studies suggest that a retrogasserian is preferred for its lower complication rate [24, 25]. Park et al. [15] suggest that a cumulative dose on the edge of the brainstem over 12 Gy is correlated with higher likelihood of post-SRS trigeminal deficit. The under-reporting of dosimetric parameters in relation to the brainstem or the surrounding medial temporal lobe is a crucial limitation in current literature: from what emerges from our analysis, there is a general sensibility on the exposure of OARs, but it remains a highly subjective procedure.

### Clinical Outcome

Overall, we found that repeat SRS is a viable option in recurrent/refractory TN, with 73% of patients achieving a BNI ≤ III. While most authors report rates of efficacy over 70%, some other cases were not so favourable. As previously indicated, moderate variability was found in data reporting among studies (I² = 67.1%, *p* < 0.001).

In particular, Tempel et al. [16] show a mere 47% of patients who responded to a second cycle of SRS. It has to be noted that Tempel’s study was centred on patients reaching a third cycle of SRS, thus selecting patients that recurred or did not respond to the first two cycles. Moreover, they reported 18 procedures before the first SRS treatment, 2 rhizotomies between the first and the second cycle, and 4 rhizotomies between the second and the third, suggesting a particularly generic subpopulation with resistant TN. Other authors [9] pinpointed that patients with poor responses after initial SRS reported poor outcomes after repeat SRS. Accordingly, our analysis showed that patients who responded to the first cycle of SRS (BNI ≤ III at initial post-SRS evaluation) are six times more likely to have a favourable outcome after a second SRS treatment (OR 6.0, CI 95%: 1.34–26.81). This evidence might suggest that patients with recurring TN might benefit more from a repeated SRS treatment rather than patients with refractory TN, but data on these two entities were not individually extractible in selected studies and separate analysis could not be performed. Tempel also reported a relatively short history of pre-SRS symptoms - mean 20 months – against longer history highlighted by other authors, such as Hasegawa et al. [8] and Brisman et al. [9] who achieved sensibly higher rates of favourable outcome (respectively 85% and 78%). This might suggest that the longer the pre-SRS symptoms, the better the response. Notably, Omar et al. [17] shown a 58% efficacy of repeat SRS even though their series had a mean 123.4 months-old history of pre-SRS symptoms.

Eventually, since 101 patients out of 428 reported (23.6%) experienced another recurrence after a second SRS treatment, about 50% of patients undergoing repeat SRS for recurrent or refractory TN have a favourable outcome.

While MS is a known negative prognostic factor in TN responsiveness to traditional treatments [26, 27], Franzini et al. [19] reported 83% with recurrent/refractory TN suffering from MS achieving a BNI ≤ III, all with typical TN. Of those 33% relapsed after a mean 12 months. Eventually, 50% of patients responded to repeat SRS treatment even if MS-associated. SRS has shown promising results in patients with MS-associated TN in Herman et al.’s series, too [9].

Taking into account pre-SRS treatment, three authors [13, 18, 19] firstly referred their patients to SRS: of the 64 patients considered, 48 (75%) achieved a BNI ≤ III after the first treatment, and 44 (64.7%) after the second. On the other hand, in eight studies [8–10, 12, 14–17] patients had already undergone an invasive procedure for the treatment of TN before the first SRS cycle. In these cases, 227 out of 299 (75.9%) patients showed a favourable outcome after the first SRS sitting, and 239 out of 359 (66.6%) after the second one. These percentages seem overall comparable to those of patients who underwent SRS as their first treatment, suggesting that radiosurgery efficacy isn’t influenced by previous non-medical treatments.

Mean re-irradiation time spanned 19.6 months (range 8–45) against a mean recurrence time of 25.9 months. While this is counter intuitive, it is probably due to the under-reporting of the recurrence time, with only 6 out of 13 studies [8, 11, 15, 16, 18, 19] mentioning it. Nonetheless, this evidence remarks the short-term recurrences [28] patients affected by TN experience, hence the extreme importance in properly assessing the adequate interval time in-between SRS cycles.

### Complications

Data extracted from the considered studies confirms the safety of repeated SRS for TN. The main complication regarded the V cranial nerve: out of 461 patients included, 202 (43.8%) suffered some degree of facial sensory impairment after two or more sittings of SRS. While for the most part the symptom was mild, 6 patients (1%) developed “anesthesia dolorosa”. Huang et al. [13] individuated 120 Gy as the limit over which facial numbness is developed (*p* < 0.037). Accordingly, before 2010, only 23.5% developed hypoesthesia, while after 2010, when higher doses have been implemented as previously stated, 48.7% of patients experienced different level of hypoesthesia after repeated SRS. Nonetheless the patients described facial numbness preferable to the original trigeminal pain, even when somewhat bothersome [7]. Shetter et al. [7] also described how patients with facial numbness had a greater likelihood of being pain free than those with no sensory loss.

Only Helis et al. [14] described complications other than V cranial nerve disfunction: 10 patients suffered from corneal dryness (6%) and 1 from taste loss (0.6%), likely from VII cranial nerve involvement. No cases of radionecrosis, hydrocephalus, or brainstem damage were described.

### Limitations

The main limitation of this study is the retrospective nature of the studies included. Risk of bias assessment [Fig. 4] individuated 2 studies with high risk of bias because they did not report recurrence after the second SRS cycle.

Another limitation resides in the heterogeneity of data reported. Nevertheless, our results achieved statistical significance.

## Conclusions

Repeat SRS is efficient in controlling pain in recurrent or refractory TN and can be repeated even in a short time span. Roughly 50% of patients have their pain controlled by a repeat SRS treatment. Patients can be reirradiated multiple times after a relatively short time span with a 43.8% chance of developing some degree of V cranial nerve disfunction and negligible cases of radionecrosis, hydrocephalus, or damage to the brainstem, even if the target isn’t modified between cycles.

Overall, SRS appears as a safe and effective compromise considering it takes one sitting of treatment repeated in time to control pain in half the patients who are often severely compromised by the condition (especially if compared to the complication rate as opposed to more invasive treatment options).

Despite the favorable premises, there’s lack of common treatment protocols and of data reporting, e.g. accurate dosimetric details of the treatments in terms of target delineation and dose constraints of OARs for repeat SRS. Further standardized and rigorous studies are needed to fully unveil SRS’s full potential in TN management algorithm.

## Data Availability

No datasets were generated or analysed during the current study.
